# CD19 chimeric antigen receptor T-cell therapy in adult patients with Philadelphia chromosome-positive acute lymphoblastic leukemia without complete molecular response at 3 months

**DOI:** 10.1038/s41408-023-00848-0

**Published:** 2023-05-10

**Authors:** Zhenzhen Yao, Bin Gu, Jia Chen, Yang Xu, Feng Chen, Shengli Xue, Huiying Qiu, Xiaowen Tang, Yue Han, Suning Chen, Aining Sun, Lei Yu, Yanming Zhang, Depei Wu, Ying Wang

**Affiliations:** 1 National Clinical Research Center for Hematologic Diseases, Jiangsu Institute of Hematology, The First Affiliated Hospital of Soochow University, Suzhou, China; 2 Institute of Blood and Marrow Transplantation, Collaborative Innovation Center of Hematology, Soochow University, Suzhou, China; 3 Key Laboratory of Thrombosis and Hemostasis of Ministry of Health, Suzhou, China; 4 Shanghai Unicar-Therapy Bio-medicine Technology Co., Ltd, Shanghai, China; 5 Department of Hematology, The Affiliated Huai’an Hospital of Xuzhou Medical University and The Second People’s Hospital of Huai’an, Huai’an, China; 6 State Key Laboratory of Radiation Medicine and Protection, Soochow University, Suzhou, China

**Keywords:** Acute lymphocytic leukaemia, Immunotherapy

**To the editor:**

Philadelphia chromosome-positive acute lymphoblastic leukemia (Ph+ ALL) is the most common subtype of ALL in adults and is historically associated with worse outcomes. While the landscape has significantly improved with the introduction of tyrosine kinase inhibitors (TKIs), relapse remains a significant clinical challenge [1]. Assessment of measurable residual disease (MRD) by reverse transcriptase polymerase chain reaction (RT-PCR) has been proven to be a powerful prognostic tool that allows better stratification of patients. Early studies showed that failure to achieve complete molecular response (CMR) at 3 months could be a warning sign of treatment failure and predicted a poor long-term outcome [2, 3]. Novel treatment modalities are needed for this high-risk subset of patients.

The advent of CD19 chimeric antigen receptor (CAR) T-cell therapeutics offers a highly promising treatment option for relapsed/refractory (r/r) B-cell ALL, including Ph+ ALL [4, 5]. Data indicate that very high rates of response can be achieved and some patients may be cured without the need for allogeneic hematopoietic stem cell transplantation (allo-HSCT). However, the role of CD19 CAR T-cell therapy in patients not achieving 3-month CMR has not been reported. Herein, from a phase 2 prospective, single-arm study of CD19-specific CAR T-cell therapy for r/r B-ALL (clinical trial NCT03919240), we report the outcomes of 13 Ph+ ALL patients in their first complete remission (CR) without 3-month CMR who were treated with CD19 CAR T-cell therapy.

CAR T-cells were manufactured by Unicar-Therapy Bio-Medicine Technology Co. (Shanghai, China), using a CAR construct comprising a 4-1BB costimulatory domain as previously described [6]. Lymphodepletion with fludarabine and cyclophosphamide was conducted prior to a split-dose escalation infusion of 5 × 10^6^ CAR-positive T cells/kg in 2–3 days. TKI therapy was suspended before the infusion of CAR T cells and resumed at least 1 month after infusion at the discretion of the treating physician. Hematologic and molecular responses were assessed by bone marrow aspirate. CMR was defined as the absence of a detectable BCR-ABL1 transcript by RT-PCR with a sensitivity of 0.01%. The study was conducted in accordance with the principles of the Declaration of Helsinki and was approved by the independent ethics committee of the First Affiliated Hospital of Soochow University. Written informed consent was provided by all patients.

From Jan 01, 2017 to Sep 30, 2021, 13 patients were identified and included in this study. The patients’ clinical characteristics are shown in Table 1. The median age was 32 years (range 26-57). Overall, the treatment was well-tolerated and all side effects were reversible. Cytokine release syndrome (CRS) occurred in 6 (46.2%) patients, and grade 3 CRS occurred in 2 patients, each received dexamethasone and tocilizumab with subsequent resolution. The median duration of CRS was 3 days (1–9). Only one grade 1 neurological events were observed. The median peak expansion day and proliferation level were 7 days (1–14) after the last infusion and 6.79 × 10^4^ copies/μg genomic DNA (1.47 × 10^4^–7.50 × 10^6^), respectively (Fig. 1A). CMR was achieved in 9 patients at Day 30 after infusion. Nine patients received allo-HSCT at the discretion of the patient or treating physician. The median time to allo-HSCT was 53 days (35-106) after CAR T-cell infusion. All patients achieved engraftment with full donor chimerism and CMR. Two patients died of infection after transplant, but no patient relapsed. Four patients (3 with CMR) who did not receive allo-HSCT received only TKI maintenance therapy and central nervous system prophylaxis with lumbar punctures. Two patients were still in ongoing CMR, while 2 patients had CD19 relapse. One patient achieved remission after CD19/CD22 dual-antigen CAR T-cell therapy and is alive at the last follow-up. The median follow-up of the living patients was 23.5 months (range, 14-63). The median event-free survival for the whole cohort were 23 months (range 2-64). An overview of the patients’ clinical courses is shown in Fig. 1B.

**Table 1:** Patient characteristics.

Number	Gender (M/F)	Age	Karyotype (additional abnormality)	Gene mutation	BCR-ABL1 transcript	WBC count at diagnosis (×10^9^/L)	Types of previous TKIs	BCR/ABL copies / 10,000 ABL copies before CAR T-cell therapy	CRS grades	BCR/ABL copies / 10,000 ABL copies at Day 30 after CAR T-cell therapy	Time from CAR T-cell infusion to allo-HSCT (days)	Donor type
1	M	34	none	none	P210	387.6	Dasatinib	1277	3	330	51	MUD
2	F	53	complex	ETV6	P210	44.6	Dasatinib	70	0	0	48	haplo
3	M	32	none	none	P190	2.5	Dasatinib	12	1	0	54	haplo
4	F	46	none	none	P190	7.7	Nilotinib	306	1	0	98	haplo
5	M	26	der(20)	none	P190	491.1	Dasatinib	14	0	566	53	haplo
6	M	27	none	none	P190	11.4	Dasatinib	6	0	0	35	haplo
7	F	28	+22, +Ph	BCORL1	P190	67.2	Imatinib	49	3	0	35	haplo
8	M	46	complex	DNMT3A, FLT3	P210	532.8	Dasatinib	96	0	0	57	haplo
9	M	50	-7	EZH2, EGFR	P190	100.3	Dasatinib	185	1	184	106	haplo
10	F	57	none	KMT2C	P190	24. 4	Dasatinib/flumatinib	30	0	0	—	—
11	F	32	-7	ASX1	P210	39.4	Dasatinib	296	0	0	—	—
12	F	29	none	GATA1, SETD2	P210	128.1	Imatinib	2123	1	0	—	—
13	F	27	complex	none	P190	20.1	Imatinib/flumatinib	18	0	27	—	—

*M* male, *F* female, *Ph* Philadelphia chromosome, *WBC* white blood cell, *TKIs* tyrosine kinase inhibitors, *CRS* cytokine release syndrome, *MUD* matched unrelated donor, *haplo* haploidentical.

**Fig. 1:**
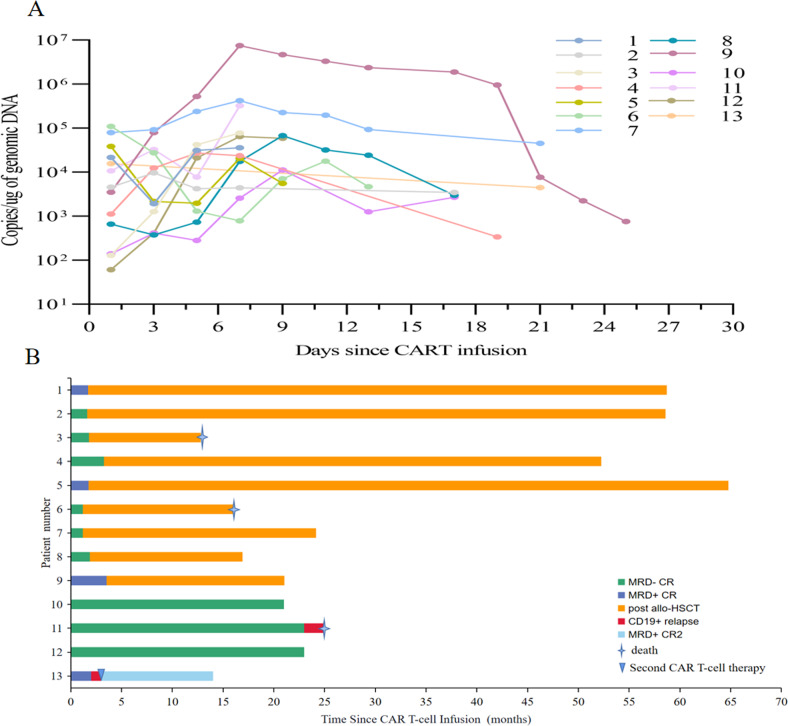
CAR-T cells expansion and outcomes for patients. **A** The CAR T-cell DNA levels in peripheral blood. **B** Swimmer plots of the 13 patients.

Non-CMR at 3 months in patients with Ph+ ALL was independently associated with worse survival and may identify a subgroup of patients who, when eligible, should be considered for consolidation with allo-HSCT [2]. As higher MRD levels prior to HSCT were predictive factors for a higher cumulative incidence of relapse, novel salvage treatments to eradicate MRD before transplantation are essential to improve the outcomes after allo-HSCT.

CAR T-cell therapy has been shown to produce high remission rates in patients with r/r B-cell ALL, but relapse is still an unresolved clinical challenge. Generally, it serves as a bridge to allo-HSCT. It is of great interest whether early incorporation of CAR T-cell therapy into frontline treatment could benefit patients. As disease burden is an important parameter for the outcome of CAR T-cell therapy, the scenario in patients with MRD may be different from that in patients with r/r ALL. First, similar to our data on mild toxicity, long-term follow-up of CD19-targeting (19-28z) CAR T-cell therapy, as well as a multi-institutional study of commercial tisagenlecleucel, both demonstrated that low disease burden before infusion is associated with decreased toxicity [4, 7]. Therefore, the low rate of infusion-related toxicity supports CAR T-cell therapy as a safe practice in this setting. Second and more importantly, disease burden may affect the response rate. Most previous studies have shown that low disease burden is positively associated with response [4, 7]. However, the CD19 antigen load at the time of CAR T-cell infusion correlated with the magnitude and peak engraftment of CAR T cells in one study [8]. The relatively low CMR achievement (9/13) in this study also raised concerns about the effectiveness of CAR T-cell therapy in patients with low-positive MRD. Therefore, more data are needed to elucidate the efficacy of CAR T-cell therapy in this situation.

Two of the three patients with CMR who did not receive allo-HSCT achieved durable remission in our study. These findings are in line with former studies showing that CAR T-cell therapy has the potential for cure in a subset of patients and challenge the role of allo-HSCT. In fact, besides CAR T-cell therapy, other novel immunotherapies, including blinatumomab and inotuzumab ozogamicin, are also effective in the setting of low MRD status [9, 10]. Compared with CAR T-cell therapy, they may offer a safer option in this scenario. Blinatumomab is approved for the treatment of MRD-positive Ph negative B-ALL patients since 2018, but not yet for Ph+ ALL patients. As the field continues to evolve rapidly, upfront administration of more potent TKI (such as ponatinib), together with these novel immunotherapies during the induction and consolidation phases, CMR could be achieved in the majority of patients and our reliance on allo-HSCT surely will be reduced.

In summary, though limited by the relatively small sample size in this study, our results strongly suggest that anti-CD19 CAR T-cell therapy is a safe therapeutic strategy with potential benefit for Ph+ ALL patients without 3-month CMR.

## Supplementary information

checklist

## Data Availability

The data are available from the corresponding author upon reasonable request.
